# Transcatheter Valve-in-Band Implantation in a Swine Model of Failed Mitral Repair: A Technical Note and Proof of Feasibility

**DOI:** 10.3390/jcm15197733

**Published:** 2026-10-08

**Authors:** Julia E. Katter, Jonathan Martinez Rivera, Meghan A. Matlack, Daniel A. Anderson, Emily K. Leva, Allison M. Tomasino, Henry J. Tannous

**Affiliations:** 1 Geisel School of Medicine at Dartmouth College, Hanover, NH 03755, USA; julia.e.katter.med@dartmouth.edu (J.E.K.); jonathan.martinez.rivera.med@dartmouth.edu (J.M.R.); 2 Dartmouth-Hitchcock Medical Center, Lebanon, NH 03756, USA; meghan.a.matlack@hitchcock.org (M.A.M.); daniel.a.anderson@hitchcock.org (D.A.A.); emily.k.leva@hitchcock.org (E.K.L.); 3 Center for Comparative Medicine and Research (CCMR), Dartmouth College, Lebanon, NH 03756, USA; allison.m.tomasino@dartmouth.edu

**Keywords:** transcatheter mitral valve replacement, valve-in-band, mitral annuloplasty, failed mitral valve repair, mitral regurgitation, swine model, structural heart intervention

## Abstract

Surgical mitral valve repair is the gold standard for severe primary mitral regurgitation (MR), yet when repairs fail, open reintervention carries substantial perioperative risk. Following band annuloplasty, minimally invasive options, such as transcatheter mitral valve replacement (TMVR), remain largely unexplored due to a hypothetical concern around the lack of circumferential radial support. Although transapical transcatheter mitral valve-in-band (ViB) replacement has been reported in a single human case, we report the first controlled large-animal model of ViB. A 68 kg, 20-month-old female Yucatan mini pig underwent placement of a 28 mm mitral annuloplasty band, oversized to create MR, via clamshell sternotomy under cardiopulmonary bypass; a simulated dilated left atrium enabled transcatheter access. A 23 mm Edwards SAPIEN 3 Ultra valve was deployed within the band using the Commander delivery system under fluoroscopic guidance. The valve achieved stable fixation without embolization, paravalvular leak (PVL), or left ventricular outflow tract (LVOT) obstruction with resolution of MR on echocardiography. The stable position was consistent with a contribution from the native anterior leaflet and annulus, although radial forces were not directly measured. The animal tolerated the procedure without complications. This first-in-animal study suggests the acute technical feasibility of TMVR within a mitral annuloplasty band and supports further investigation of percutaneous band modification strategies to optimize ViB outcomes.

## 1. Introduction

Mitral regurgitation (MR) is among the most common valvular lesions, with the prevalence of clinically significant valve disease rising with age, reaching 13.3% in those aged ≥75 years [1]. Surgical mitral valve repair is the gold standard for severe primary MR, offering superior long-term outcomes to valve replacement [2], including lower operative mortality (3.9% versus 8.9%) and superior 1-, 5-, and 10-year survival [3,4]. At high volume centers, roughly three-quarters of degenerative valves are repaired rather than replaced [5]. Repair is completed with an annuloplasty device, either a complete ring or a partial band, and device choice varies by surgeon and center. Partial posterior bands, favored to preserve anterior annular and leaflet mobility and the physiologic saddle-shaped dynamics of the annulus, predominate at some centers (roughly two-thirds of repairs in one series [6]), whereas complete rings remain the majority for degenerative mitral repair in the United States [7]. Repairs are durable but not always permanent. They can fail through a variety of mechanisms, including incomplete correction of MR at the baseline operation, continued disease progression, or leaflet calcification [8]. Significant regurgitation recurs progressively in the years after surgery [9], affecting approximately 16.5% of patients with reported reoperation rates ranging from 4.5–10% [10]. Because repair volumes are large and many affected patients are elderly or otherwise poor candidates for redo sternotomy, a substantial and growing population lives with a failing repair, yet minimally invasive options to avoid redo sternotomy remain limited.

For patients who are poor surgical candidates, transcatheter mitral valve replacement (TMVR) has emerged as a reintervention strategy, with established valve-in-valve (ViV) and valve-in-ring (ViR) options for failed bioprostheses and complete rings. Yet, for failed bands, valve-in-band (ViB) intervention remains largely unexplored, limited to an isolated case report demonstrating a transapical ViB approach [11], with no US Food and Drug Administration (FDA)-approved option. The principal concern for valve-in-band is incomplete annular support; partial bands reinforce only the posterior annulus, leaving the anterior annulus unsupported and theoretically predisposing to insufficient fixation, embolization, left ventricular outflow tract (LVOT) obstruction, and paravalvular leak (PVL) [12]. Although the expanding valve-in-ring experience offers transferable lessons in device selection, sizing, and pre-procedural LVOT-obstruction screening, no comparable framework yet exists for valve-in-band. The aim of our work is to begin building such a framework. Our model is part of a broader research program (IACUC Protocol #00002378) evaluating percutaneous ring completion and transcatheter valve deployment following atrioventricular-valve band placement in both the mitral and tricuspid positions. Building on a swine model that recreates failed repair by generating MR within an annuloplasty band, we deployed a TMVR device within the band in a large animal.

## 2. Materials and Methods

Study design and animal. This was a single-animal, non-survival, first-in-animal feasibility study conducted under a protocol approved by the Dartmouth College Institutional Animal Care and Use Committee (IACUC Protocol #00002378; approved 4 November 2025; Animal Welfare Assurance D16-00166). The study protocol, including its objectives and design, was defined a priori and approved by the IACUC; it was not entered in a public registry. Reporting follows the ARRIVE 2.0 guidelines. One 20-month-old, 68 kg female Yucatan mini pig (Sinclair Bio Resources, Auxvasse, MO, USA) was studied and was housed and acclimated in the Dartmouth College Center for Comparative Medicine and Research. A single animal was used because the objective was proof of technical feasibility rather than a statistically powered comparison. The experimental unit was the individual animal; no control group was included, and no a priori sample-size calculation was performed, consistent with a single-arm feasibility design. Randomization, blinding, and formal inclusion/exclusion criteria were not applicable to a single-animal study, and there were no exclusions. The animal was healthy at enrollment, with no prior experimental procedures.

Anesthesia and monitoring. Following a 12 h fast, the animal was premedicated with intramuscular tiletamine-zolazepam (Telazol, 4.4 mL) and xylazine (0.44 mL), intubated (7.5 mm endotracheal tube), and maintained on isoflurane (2–3%) in oxygen (2 L/min). Neuromuscular blockade was provided with rocuronium, and heparin, calcium chloride, magnesium sulfate, lidocaine, and sodium bicarbonate were administered intravenously as indicated. Monitoring included pulse oximetry, end-tidal CO_2_, invasive arterial pressure, temperature, and serial arterial blood gases. Anesthesia and analgesia were titrated to minimize pain and distress; as a terminal procedure performed entirely under general anesthesia, the study had no humane endpoints or recovery-monitoring schedule, and no unexpected adverse events occurred.

Surgical preparation and cardiopulmonary bypass. The animal was aseptically prepped and draped in the hybrid operating suite (Center for Surgical Innovation, Lebanon, NH, USA). The chest was opened via clamshell incision with division of the internal mammary arteries; to maximize exposure, the sternum and ribs were divided to approximately the midaxillary line, a retractor was placed, and the pericardium was opened. A hypoplastic aorta (~1.5 cm) was noted without other structural abnormalities. After heparinization to an activated clotting time > 400 s, central cannulation was performed (16 Fr arterial, 36 Fr venous) and cardiopulmonary bypass was initiated using a Medtronic oxygenator with a Plasma-Lyte prime (~1400 mL). The aorta was cross-clamped and the heart was arrested with antegrade cold del Nido cardioplegia (4:1; ~1.9 L), with mild systemic hypothermia (~34–35 °C) during the arrest.

**Figure 1:**
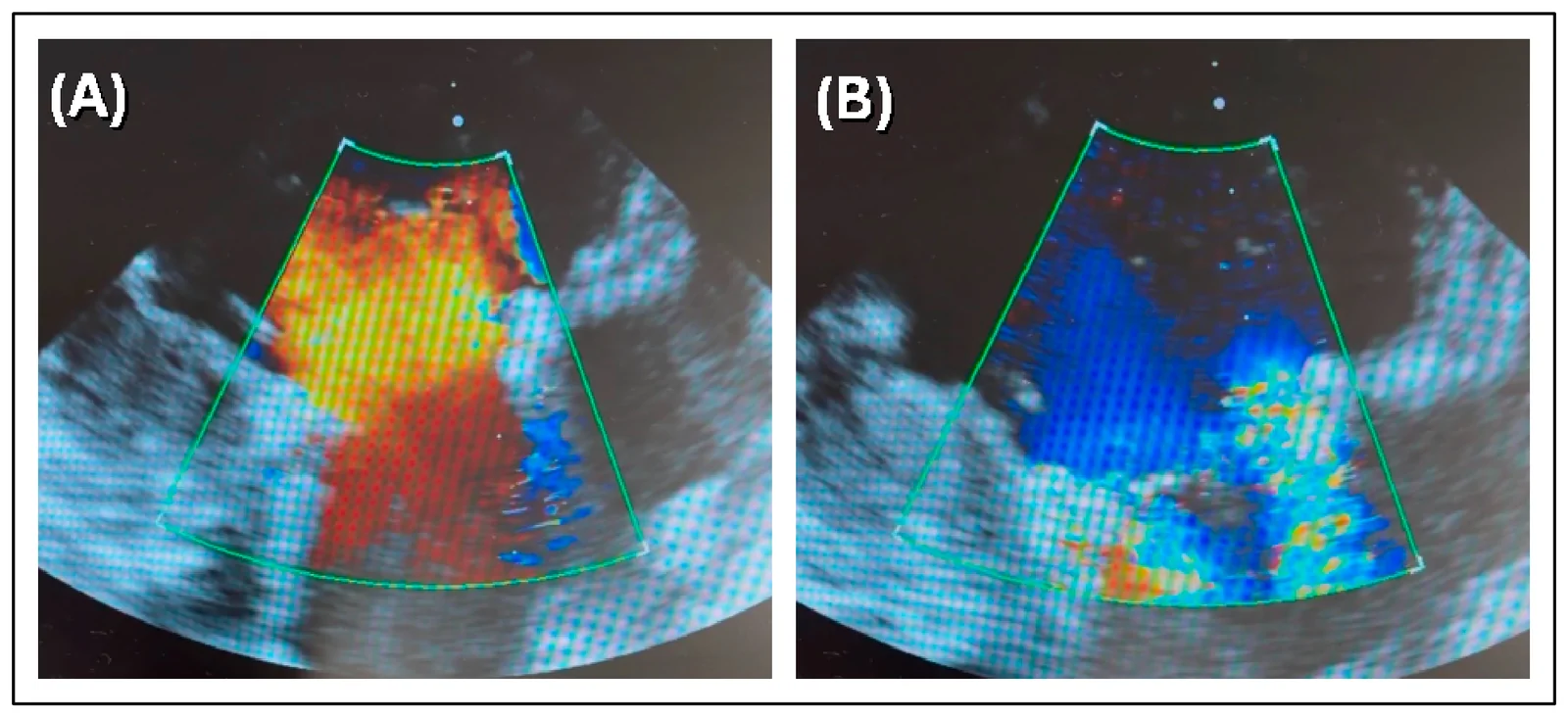
Intraoperative color Doppler echocardiography after band annuloplasty, modeling failed mitral repair. (**A**) Transmitral inflow into the left ventricle during diastole. (**B**) Broad regurgitant jet confirming severe mitral regurgitation.

Model creation. Through a left atriotomy (accessed by following the right pulmonary vein leading us to the interatrial septum), the mitral valve was exposed and circumferential 2-0 Ethibond sutures were placed around the posterior annulus from trigone to trigone. A 28 mm SimuPlus flexible mitral annuloplasty band (Model 7700FB, Medtronic, Minneapolis, MN, USA), selected 2 mm larger than the 26 mm size indicated by a standard annuloplasty sizer to generate mitral regurgitation, was seated and secured with core knots (Figure 1). To enable subsequent transcatheter access, a Gelweave mesh conduit with an 8 mm side-arm was anastomosed to the left atrium to simulate a dilated atrium (needed to better maneuver the valve in a small atrium). The cross-clamp was removed after 75 min of ischemia and the heart resumed sinus rhythm.

**Figure 2:**
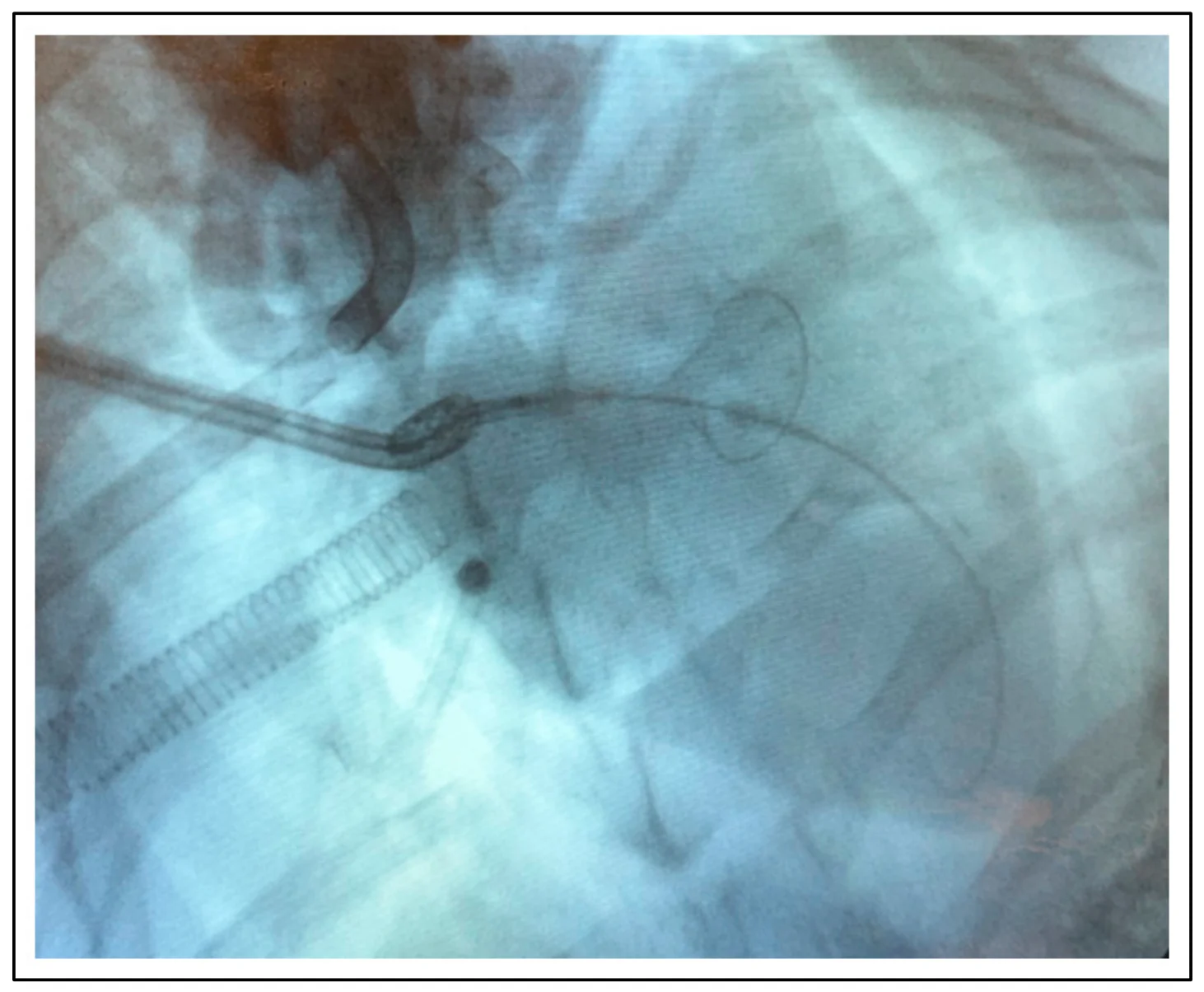
Fluoroscopic image of transcatheter access. The sheath enters through the left atrial graft, and the guidewire crosses the mitral valve and curves within the left ventricle.

**Figure 3:**
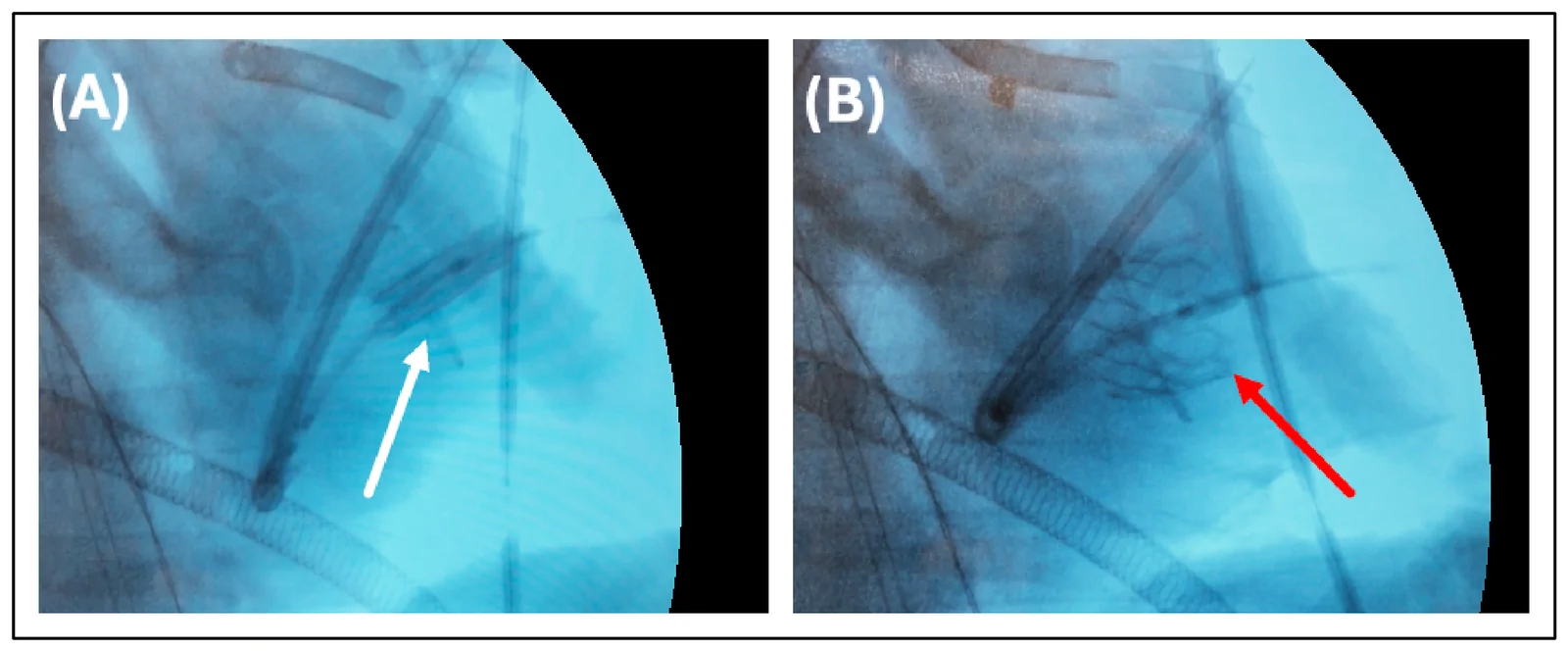
Fluoroscopic images of transcatheter valve deployment within the mitral annuloplasty band. (**A**) Pre-deployment positioning of the crimped valve within the band (white arrow). (**B**) Post-deployment image showing the expanded valve frame within the band (red arrow).

Device selection and transcatheter deployment. A 23 mm Edwards SAPIEN 3 Ultra (Edwards Lifesciences, Irvine, CA, USA) was selected on the basis of a qualitative ex vivo assessment of the implanted 28 mm partial band and sizing. This assessment was preliminary and experiment-specific, and the band internal diameter and effective anchoring dimension were not measured with a standardized, reported technique. The nominal deployed outer diameter of a 23 mm balloon-expandable valve is approximately 22.7 mm at the inflow plane on ex vivo bench testing [13]. At nominal volume, the frame was therefore undersized relative to the 28 mm labeled band. Manufacturer sizing charts are defined for a native annulus or a failing bioprosthesis rather than for an annuloplasty band [14]. With the animal on partial cardiopulmonary bypass for hemodynamic support (pump flow approximately 1.4–1.7 L/min, versus 3–4 L/min on full bypass), guidewire access to the left ventricle was established through the left atrial graft under fluoroscopic guidance (Figure 2), and the valve was deployed within the band using the Commander delivery system (Figure 3). The balloon was inflated with 19 mL, overfilled by 2 mL above the manufacturer’s nominal volume [14,15], until the frame was visually apposed to the band, under rapid ventricular pacing at 140 beats/min. Fluoroscopy and echocardiography guided and documented positioning.

Assessments and euthanasia. Endpoints were defined according to the Mitral Valve Academic Research Consortium (MVARC) framework [16]. Per MVARC, the primary outcome was technical success, defined as successful access, delivery, and deployment of the valve within the band in the intended position with retrieval of the delivery system, and freedom from procedural death or emergency surgery or reintervention. MVARC technical success was developed for percutaneous, transseptal access. In this model, the access and delivery components were satisfied through the surgically constructed left atrial conduit under open-chest cardiopulmonary bypass rather than by transseptal catheter access. This adaptation limits direct comparability with percutaneous human series. Residual mitral regurgitation was graded on the MVARC ordinal scale (none or trace, mild, moderate, severe). Additional secondary outcomes included paravalvular leak, LVOT obstruction, valve thrombus, and periprocedural survival. Valve position, apposition, mitral regurgitation, paravalvular leak, thrombus, and left ventricular outflow tract patency were assessed by intraoperative echocardiography with color Doppler and corroborated by direct inspection and postmortem dissection. Given the single-animal design, outcomes are reported descriptively, without formal statistical analysis. At completion of this non-survival protocol, euthanasia was performed by the attending veterinarian with intravenous pentobarbital-phenytoin (Euthasol), and death was confirmed by the absence of cardiac function. MVARC device success, assessed at 30 days, requires proper device positioning, no procedural mortality, stroke, or device-related reintervention, reduction in MR without significant mitral stenosis, and no more than mild paravalvular leak [16]. It could not be assessed in this acute, non-survival design.

## 3. Results

**Figure 4:**
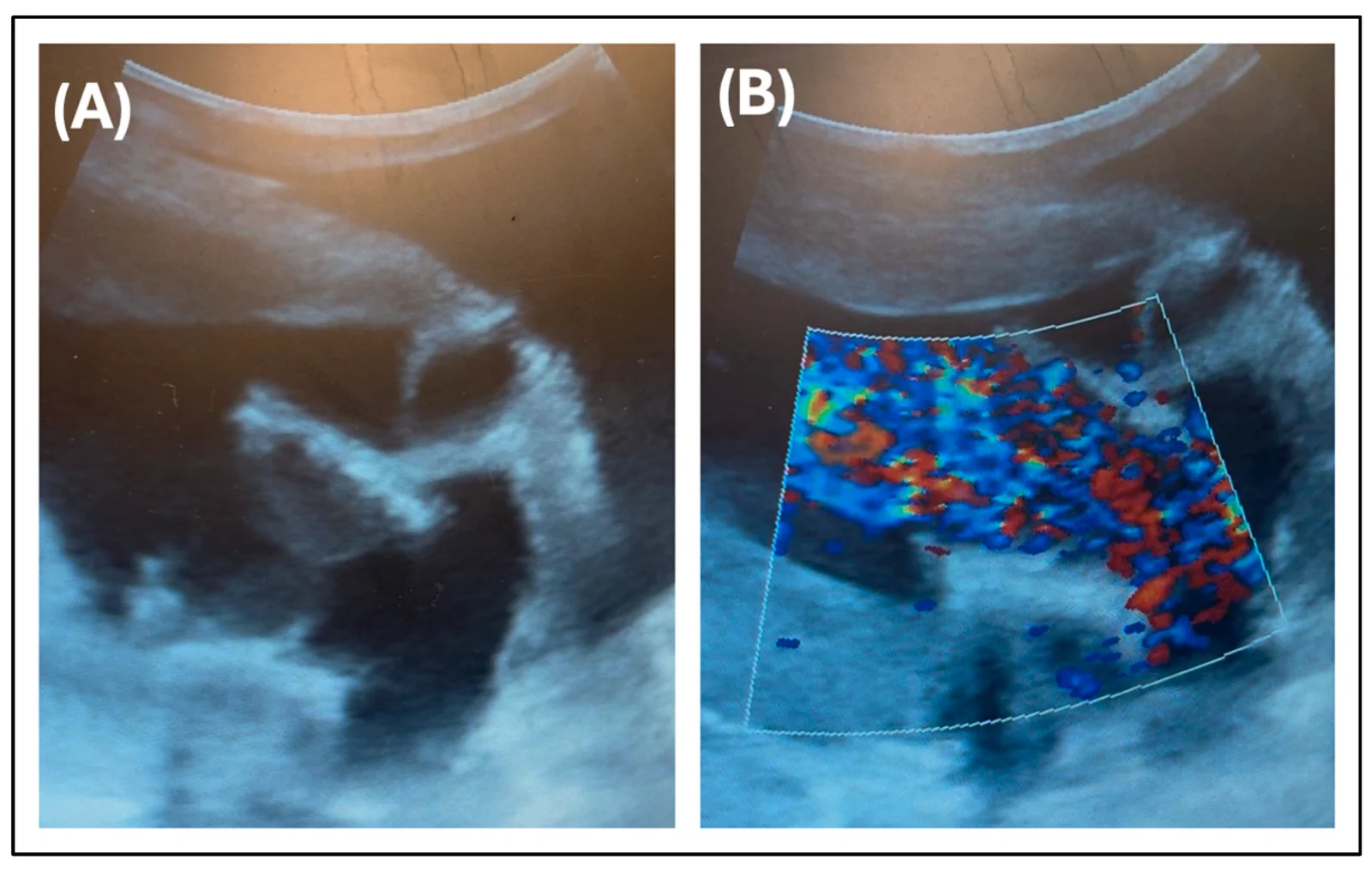
Intraoperative echocardiography after valve deployment, long-axis view. (**A**) B-mode image showing the valve frame seated within the band and its relationship to the native anterior leaflet. (**B**) Color Doppler image of the same view showing transvalvular inflow without a regurgitant or paravalvular jet.

The animal tolerated the procedure without perioperative complications. After band placement, severe MR was confirmed by intraoperative echocardiography, and fluoroscopy confirmed correct TMVR positioning within the band. Technical success, as defined by MVARC, was achieved as defined above: the valve was accessed and deployed within the band in the intended position. Following deployment, the valve frame was stable with appropriate contact, and without redundant leaflet motion, frame prolapse, or embolization. The frame position was consistent with the native anterior leaflet and annulus complementing the posterior and lateral fixation provided by the band, although radial contributions were not directly measured. Echocardiography demonstrated a well-functioning valve with resolution of MR (MVARC grade trace or none) without thrombus or paravalvular leak on color Doppler (Figure 4). No LVOT obstruction was identified on echocardiography.

**Figure 5:**
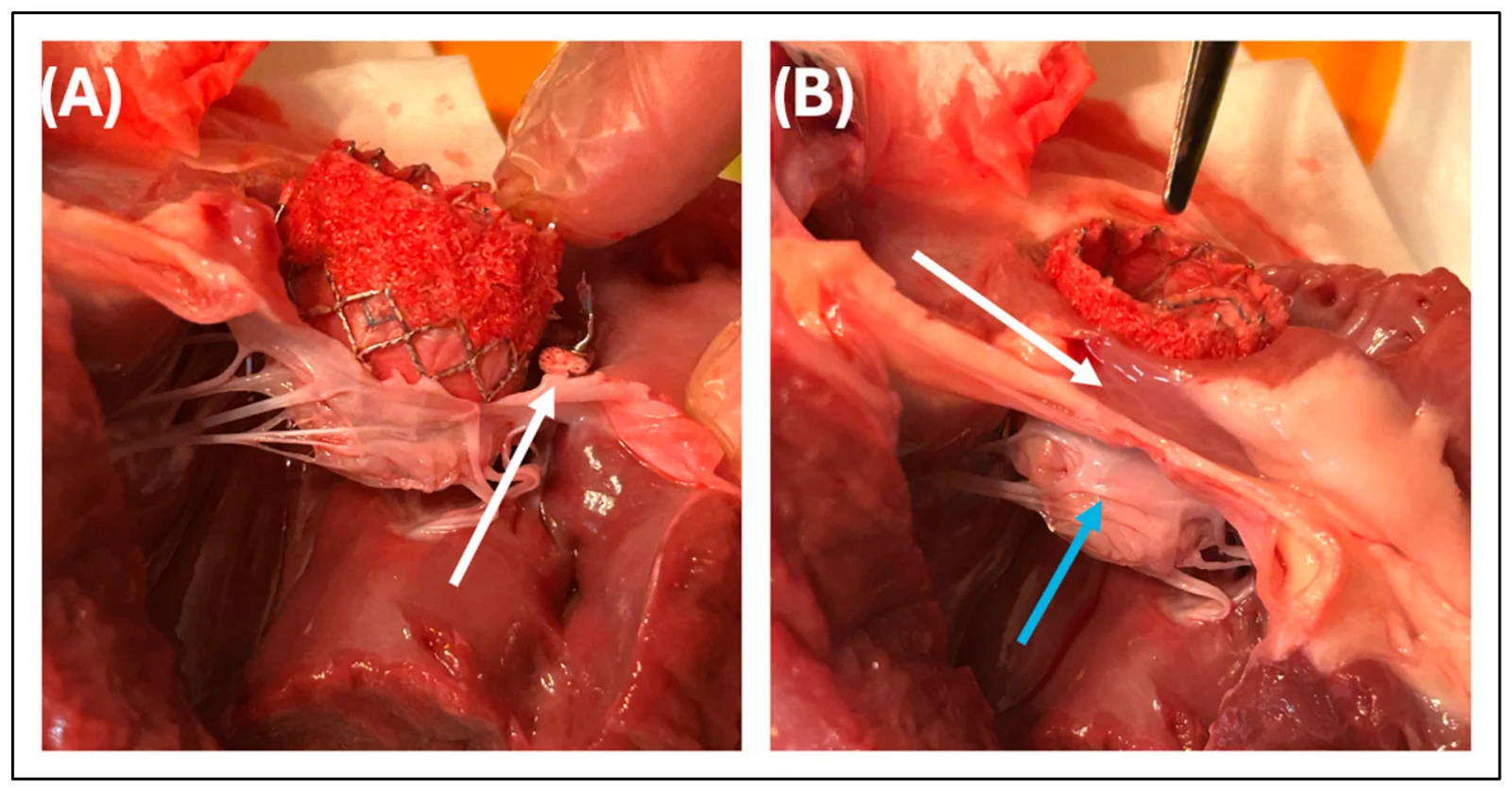
Postmortem inspection after deployment: (**A**) apposition of the transcatheter valve frame against the band near its trigonal end (white arrow); (**B**) support of the anterior leaflet and annulus (white arrow) and a patent left ventricular outflow tract without obstruction (blue arrow).

Doppler loops were not acquired at a sweep speed suitable for offline measurement, so transmitral gradient and effective orifice area were not quantified. Mean arterial pressure was approximately 55 mmHg immediately before deployment and 52 mmHg immediately afterward. Heart rate was not reliably acquired during deployment because of rapid ventricular pacing. No obstruction was evident on postmortem dissection. Direct visualization confirmed apposition of the valve frame against the band without focal gaps or LVOT obstruction (Figure 5).

## 4. Discussion

Failed partial-band mitral repair leaves a large and growing population without a minimally invasive option. A partial posterior band is often chosen because it stabilizes the dilated posterior annulus while preserving anterior leaflet and annular mobility [17], with durability comparable to a complete ring [18]. The trade-off is that structural support is confined to the posterior annulus. Valve-in-valve and valve-in-ring succeed because the bioprosthetic valve or complete ring gives the transcatheter valve a circumferential landing zone [19,20]. However, a partial band does not; thus, valve-in-band has remained mainly theoretical until this point [12]. This study sought to investigate whether a band can successfully anchor a transcatheter valve. At least acutely, the valve remained seated stably without embolization or paravalvular leak (Figure 4 and Figure 5). The stable position and postmortem appearance suggest that the preserved native anterior annulus and leaflet supplied the anterior support a partial band omits. This mechanism is inferred rather than measured: radial forces were not quantified, and the contributions of band, annulus, leaflet, ventricular geometry, and procedural conditions cannot be separated here. Even so, the acute result is encouraging and supports further formal biomechanical evaluation.

The valve-in-valve and valve-in-ring experience, though built on a different anchoring substrate, offers a template for preoperative planning and screening for valve-in-band candidates. Recent literature has found that valve-in-ring and valve-in-mitral annular calcification are feasible, but not without risk, citing 22.4% 1-year mortality, a 9.1% reintervention rate, and left ventricular outflow tract obstruction as a leading cause of death [19,20]. Longer-term data temper this further: in the MITRAL trial, 5-year all-cause mortality after mitral valve-in-ring reached approximately 65%, yet transcatheter valve function and mean gradients remained stable, indicating that the limiting factor in this population is underlying disease severity rather than device performance [21]. Also, a dedicated, fully percutaneous transseptal device now exists: the SAPIEN M3 met its 1-year primary endpoint in the ENCIRCLE trial [22] and would be a competitor to a valve-in-band approach. Valve-in-band can be considered against this platform for anatomies unsuitable for a dedicated device. Preprocedural strategies for ViR can be adapted to the ViB setting, such as preprocedural computed tomography sizing of the annulus and modeling the potential LVOT obstruction. This strategy can also assess individual anterior leaflet anatomy and flag patients at risk of obstruction [23,24] and inform whether adjunctive leaflet modification is warranted [25]. Valve-in-band can adopt this planning and screening playbook.

**Figure 6:**
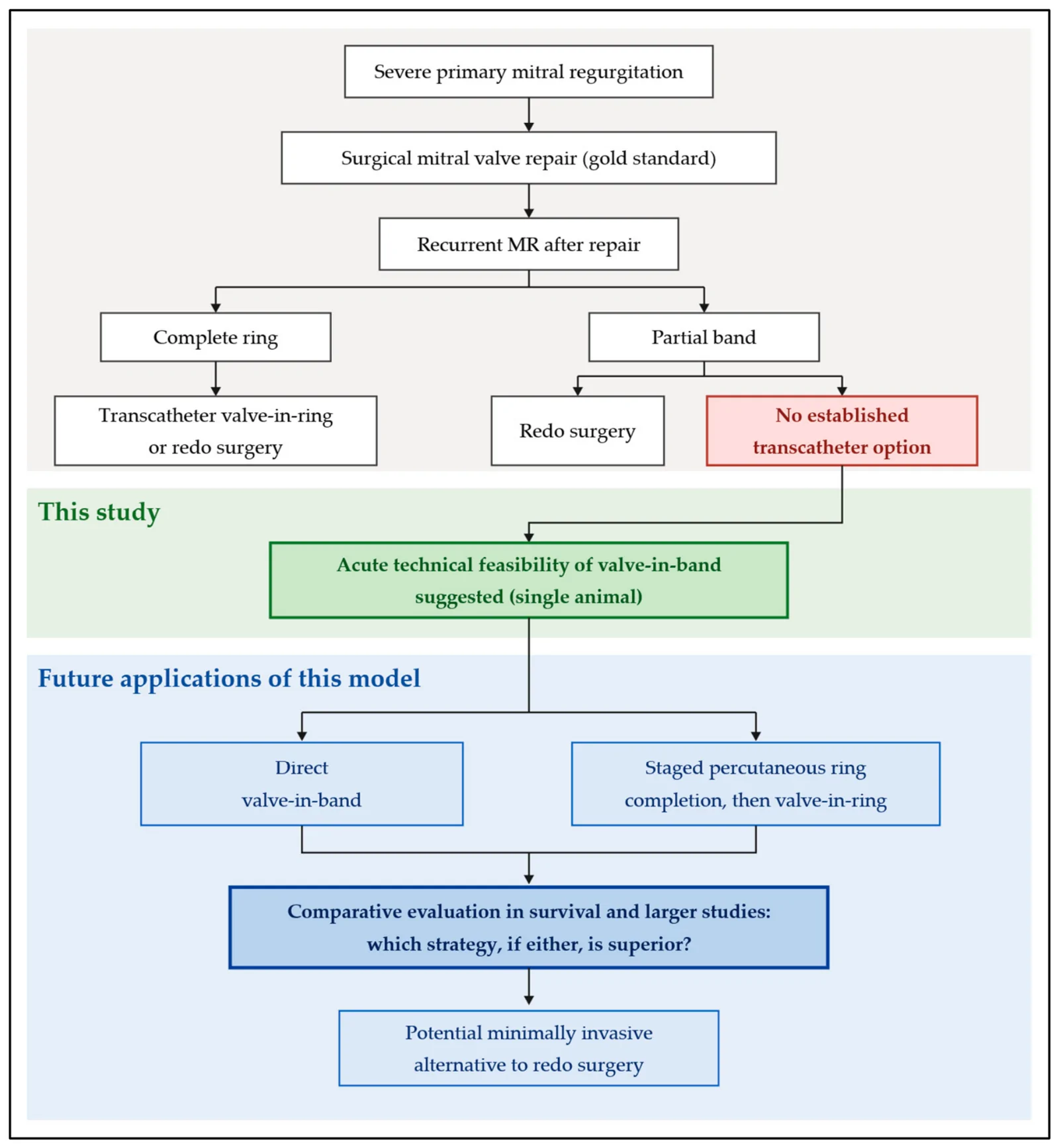
Management of recurrent mitral regurgitation after repair and proposed application of the valve-in-band model. Gray boxes indicate the established clinical pathway, red indicates the current unmet need after partial band repair, green indicates the findings of this study, and blue indicates future applications of the model.

The present experiment demonstrated acute, qualitative, single-animal deployment of a transcatheter valve within a partial band; the strategies discussed below remain hypothetical. Building on this deployment, two strategies now warrant evaluation in future studies (Figure 6). The first is direct valve-in-band, an approach approximated acutely in this experiment but not established as a percutaneous or durable procedure. The second is staged percutaneous ring completion, bridging the unsupported anterior annulus between the fibrous trigones to convert the band into a complete ring before deployment. This strategy was not performed and is proposed based on the rationale that it would restore circumferential support, reframing the procedure as the better-characterized valve-in-ring [20,23]. Comparing the two is the explicit aim of the program under which this study was performed (IACUC Protocol #00002378, approved for up to 30 animals). Definitive translation will require a fully percutaneous, transseptal approach, survival data, and adequately powered comparison against redo surgery [10] using standardized Mitral Valve Academic Research Consortium endpoints [16].

There are several important limitations to consider. This single, acute, non-survival experiment suggests technical feasibility in one anatomy under controlled operative conditions. It cannot establish reproducibility, safety, durability, or generalizability, and outcomes are descriptive rather than statistical. Chronic valve function and delayed LVOT obstruction could not be assessed. The preparation is also model-specific: partial cardiopulmonary bypass, a surgically constructed dilated left atrium for access, an incidentally hypoplastic aorta, and an experimentally oversized band all differ from a transseptal human intervention and from the chronic, heterogeneous substrate of clinical failed repair. LVOT assessment was moreover performed in an anesthetized animal on partial cardiopulmonary bypass, a low-flow, low-afterload, and anatomically atypical state. While we report the absence of apparent obstruction on intraprocedural imaging, this acute assessment cannot completely exclude the possibility of dynamic outflow-tract obstruction.

Two features of the model warrant particular emphasis. First, regurgitation was created by band oversizing, a non-degenerative mechanism with normal, fully mobile leaflets. By contrast, clinical failed repair often involves a tethered, fibrotic, or calcified anterior leaflet, the very structure to which we attribute anterior fixation. The proposed mechanism may not reproduce in real-world failed repairs. Second, band rigidity governs landing-zone behavior, so this observation applies to the flexible band used here and should not be generalized across semi-rigid and rigid designs.

Lastly, quantitative endpoints were not acquired. Transmitral gradient, effective orifice area, left ventricular and left atrial pressures, and the oversizing ratio relative to a measured band were not recorded under acute, bypass-supported conditions. Explanted-frame geometry (deformation, eccentricity, completeness of expansion) was likewise not measured, and adequate anchoring was inferred from stable positioning rather than from direct measurement. Valve selection rested on the nominal deployed frame dimension relative to the labeled band size [13], because manufacturer charts address only native annulus and bioprosthesis sizing [14]; a validated band-to-valve selection method remains to be established. Prospective, protocolized acquisition of these endpoints, together with computed tomography annular sizing and quantitative neo-LVOT and outflow-tract assessment, with transmitral gradient as the primary durability-relevant metric, is the immediate next step before any clinical translation.

## 5. Conclusions

In a single large-animal experiment of failed band annuloplasty, transcatheter valve-in-band was technically feasible, with acute procedural success, achieving stable fixation with resolution of mitral regurgitation and without embolization or paravalvular leak. The preserved native anterior leaflet and annulus are the likely source of the anterior support a partial band omits, providing a proof-of-feasibility observation that a band, long regarded as an unsuitable landing zone, can provide adequate anchoring for a transcatheter valve. These findings suggest technical feasibility in one acute experiment and establish a platform for survival and comparative studies of direct valve-in-band versus staged percutaneous ring completion, using standardized endpoints. Reproducibility, safety, durability, and generalizability remain to be established before the approach can be considered a durable, minimally invasive alternative to redo mitral surgery.

## Data Availability

The data supporting the findings of this study are available from the corresponding author upon request.

## Abbreviations

The following abbreviations are used in this manuscript:

MR	Mitral regurgitation
TMVR	Transcatheter mitral valve replacement
ViB	Valve-in-band
PVL	Paravalvular leak
ViV	Valve-in-valve
ViR	Valve-in-ring
LVOT	Left ventricular outflow tract
IACUC	Institutional Animal Care and Use Committee
FDA	US Food and Drug Administration
MVARC	Mitral Valve Academic Research Consortium
